# Real Workers in Electric Lighting

**Published:** 1882-02

**Authors:** 


### REAL WORKERS IN ELECTRIC LIGHTING.

Under this title the *Tribune* quotes from the *London Electrician* the following statement, which well expresses the exaggerated degree to which, in some quarters, the public has been led to expect impossibilities in electric lighting :

The non-technical papers, totally ignorant of the scientific aspects of the case, have introduced into the popular mind ideas altogether erroneous, yet so abiding that they still exist. The divisibility of the electric light is what the ninety and nine say the inventors are endeavoring to find, and think that, to a certain extent, success has been achieved. It is impossible to drive away the illusion and to enforce the doctrine that in this direction nothing new has been or is likely to be done. Mathematicians knew years ago as much about resistance, lamps in " seriet," or in " multiple arc," as they know now. It was not in this direction that science was working at all. The men who have developed the properties of electric lighting are those who have worked at the perfecting of the steam engine as a motor for the dynamo-machine, who have improved the dynamo and magneto machines, so that the current is more economically generated, who have experimented in every direction, and added minor improvement after improvement to the arc and incandescent lamps—these are the men who have made electric lighting not only practicable, but the light of the immediate future.—*Sanitary Engineer.*

